# Sharing the same slope: Behavioral responses of a threatened mesocarnivore to motorized and nonmotorized winter recreation

**DOI:** 10.1002/ece3.4382

**Published:** 2018-07-30

**Authors:** Lucretia E. Olson, John R. Squires, Elizabeth K. Roberts, Jacob S. Ivan, Mark Hebblewhite

**Affiliations:** ^1^ Rocky Mountain Research Station United States Forest Service Missoula Montana; ^2^ White River National Forest United States Forest Service Glenwood Springs Colorado; ^3^ Colorado Parks and Wildlife Fort Collins Colorado; ^4^ Wildlife Biology Program Department of Ecosystem and Conservation Sciences W.A. Franke College of Forestry and Conservation University of Montana Missoula Montana

**Keywords:** anthropogenic disturbance, *Lynx canadensis*, ski resorts, snowmobiles, space use, winter recreation

## Abstract

Winter recreation is a widely popular activity and is expected to increase due to changes in recreation technology and human population growth. Wildlife are frequently negatively impacted by winter recreation, however, through displacement from habitat, alteration of activity patterns, or changes in movement behavior. We studied impacts of dispersed and developed winter recreation on Canada lynx (*Lynx canadensis*) at their southwestern range periphery in Colorado, USA. We used GPS collars to track movements of 18 adult lynx over 4 years, coupled with GPS devices that logged 2,839 unique recreation tracks to provide a detailed spatial estimate of recreation intensity. We assessed changes in lynx spatial and temporal patterns in response to motorized and nonmotorized recreation, as well as differences in movement rate and path tortuosity. We found that lynx decreased their movement rate in areas with high‐intensity back‐country skiing and snowmobiling, and adjusted their temporal patterns so that they were more active at night in areas with high‐intensity recreation. We did not find consistent evidence of spatial avoidance of recreation: lynx exhibited some avoidance of areas with motorized recreation, but selected areas in close proximity to nonmotorized recreation trails. Lynx appeared to avoid high‐intensity developed ski resorts, however, especially when recreation was most intense. We conclude that lynx in our study areas did not exhibit strong negative responses to dispersed recreation, but instead altered their behavior and temporal patterns in a nuanced response to recreation, perhaps to decrease direct interactions with recreationists. However, based on observed avoidance of developed recreation, there may be a threshold of human disturbance above which lynx cannot coexist with winter recreation.

## 1 INTRODUCTION

Winter recreation is an important economic contributor to communities in temperate or subarctic regions (Töglhofer, Eigner, & Prettenthaler, 2011; White & Stynes, 2008; Zhang, Cai, & Ni, 2006). Due to technological advancements in snowmobiles, back‐country skis and other outdoor equipment coupled with growing human populations, the footprint of winter recreation continues to expand. Persistent snow‐covered areas, however, are decreasing in spatial and temporal extent due to climate change, which will likely result in increased recreation intensity in the areas that remain (Brammer, Samson, & Humphries, 2015; Elsasser & Messerli, 2001; Scott, Dawson, & Jones, 2008). Increased human disturbance to species already stressed by a changing climate may exacerbate negative effects (Hughes, 2003; Riordan & Rundel, 2014). Therefore, a better understanding of the response of animals to winter recreation in these ecosystems is critical.

While recreational use of an area is generally assumed to be more compatible with species’ conservation than consumptive activities such as development or resource extraction, animals’ perceived risk from recreation can lead to behavioral tradeoffs such as increased vigilance and decreased feeding, mating, or parental care activities (Frid & Dill, 2002; Larson, Reed, Merenlender, & Crooks, 2016). Snow‐based recreation may also have a greater negative affect on wildlife compared to aquatic or summer‐terrestrial sports (Larson et al., 2016), with changes in space or temporal use of an area frequently observed. Moose (*Alces alces*) and mountain caribou (*Rangifer tarandus caribou*), for example, were spatially displaced from suitable habitat by the presence of snowmobile recreation (Harris, Nielson, Rinaldi, & Lohuis, 2013; Seip, Johnson, & Watts, 2007), while mountain goats (*Oreamnos americanus;* Richard & Côté, 2016) and black grouse (*Tetrao tetrix*) avoided developed ski areas (Patthey, Wirthner, Signorell, & Arlettaz, 2008). Behavioral responses such as changes in activity or movement have also been observed; for instance, moose in Wyoming remained bedded or fed less frequently in response to snowmobile activity (Colescott & Gillingham, 1998).

Impacts of recreation on animals can also vary depending on whether activities are motorized or nonmotorized, dispersed or developed, and low or high intensity. While motorized recreation is frequently considered a source of disturbance (Goldstein, Poe, Suring, Nielson, & McDonald, 2010; Olliff, Legg, & Kaeding, 1999), nonmotorized recreation has also been shown to elicit negative responses in animals, and may even do so to a greater degree than motorized recreation (Harris et al., 2013; Larson et al., 2016; Stankowich, 2008). Although snowmobiles generate high noise levels, they may be perceived as less of a threat than human voices by species conditioned to fear persecution (Bowles, 1995). Additionally, many species are able to seek isolated refugia from snowmobilers, whereas nonmotorized recreationists may access remote areas with higher elevations, dense canopies, and nongroomed trails (Olson et al., 2017). Developed ski resorts, which include considerable infrastructure, tree removal, and continuous maintenance (Rixen & Rolando, 2013), also differ from dispersed recreation, which requires little infrastructure and minimally affects existing forest conditions.

The number of participants and total days spent on winter recreation is projected to increase over the next several decades (White et al., 2016), and coupled with the potential of climate‐induced reduction in persistent and deep snow, research is needed to characterize the effects of winter recreation on endangered or threatened species that are snow‐associated (Larson et al., 2016). Canada lynx (*Lynx canadensis*), a threatened species in the continental United States, is of particular concern because of its relative rarity as well as its adaptation to and reliance on deep snow to limit competition from other predators during winter (Buskirk, Ruggiero, & Krebs, 1999). Winter recreation may cause increased energy expenditure if lynx are repeatedly disturbed, as well as lost hunting opportunities since lynx are a stalking, sit‐and‐wait predator (Nellis & Keith, 1968). Western Colorado, USA is an excellent study location for this question, with an abundance of both dispersed and developed recreation, as well as a resident lynx population. The Vail Pass Winter Recreation Area in Colorado has 50 miles of established groomed trails as well as a ski‐hut system for dispersed recreation; this area is subject to intense recreation and receives roughly 35,000 visitors per winter (U.S.D.A. Forest Service 2015). Colorado also has 30 developed ski resorts (National Ski Areas Association 2016) which coincide with lynx distribution and received roughly 13 million visitors in 2016 (Blevins, 2016).

The goal of our study was to understand the impact of winter recreation on Canada lynx. We used the movement ecology paradigm (Nathan et al., 2008) to frame our investigation of winter recreation impacts on lynx behavior. Specifically, we examined (a) the impact of dispersed recreation intensity on various metrics of lynx behavior, (b) the extent to which lynx spatially and temporally avoid dispersed recreation, and (c) the extent to which lynx spatially and temporally avoid high‐intensity developed recreation. We examined behavioral metrics including movement speed and movement tortuosity that we hypothesized might be influenced by recreation and could lead to increased energy expenditure or reduction in hunting success. We hypothesized that lynx would increase speed and decrease tortuosity if disturbed by recreation, in an effort to spend as little time as possible in areas with more recreation and as a flight response to disturbance (Stewart et al., 2016). We also hypothesized that lynx would adjust their space use to avoid areas with high recreation intensity, or their temporal habits to avoid activity during high recreation‐intensity daylight hours, if disturbed by winter recreation.

## 2 METHODS

### 2.1 Study area

**Figure 1:**
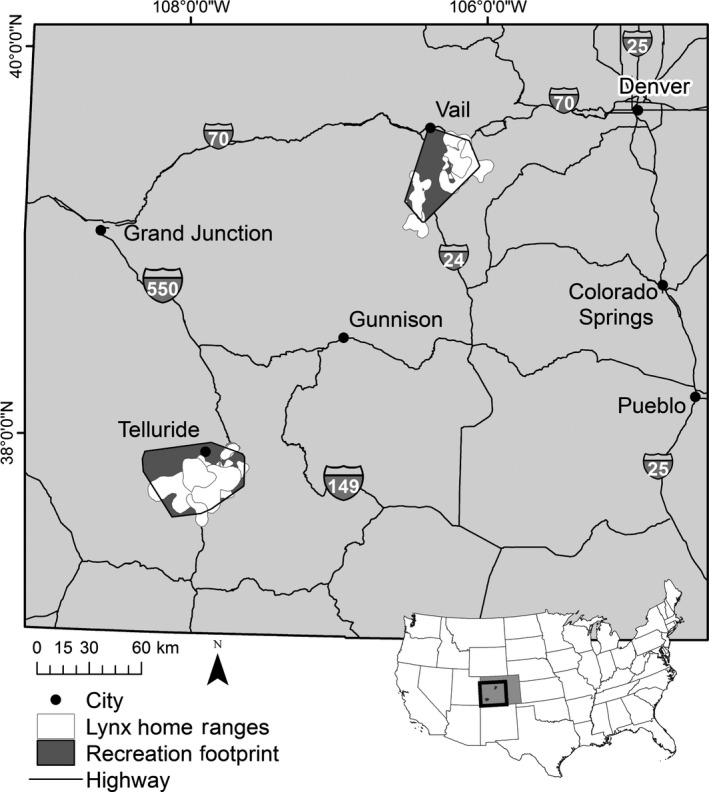
Location of Canada lynx and recreation study areas in western Colorado, USA. Canada lynx home ranges are shown in white, recreation 100% minimum convex polygons are shown in dark gray. Inset shows location of Colorado in United States

Our study area was located in western Colorado, USA, at two locations of high recreation activity (Figure 1). The northern Vail Pass study area was on public lands administered by the White River National Forest and the San Isabel National Forest, in the northern Sawatch and Mosquito mountain ranges (approximate centroid coordinates 106.30°W, 39.45°N). The San Juan study area was on public lands administered by the Uncompahgre and San Juan National Forests, and the Bureau of Land Management, in and around the towns of Silverton and Ophir (approximate centroid 107.88°W, 37.82°N). Winter recreation occurred on both sites between end of December and early April, at elevations of 2,000 m to 4,300 m and with approximately 380 cm to 1,000 cm of annual snowfall (National Oceanic and Atmospheric Adminstration 2017).

Recreation at the Vail Pass location is intense, and includes approximately 35,000 visitors per winter season, the majority of which are motorized and concentrated along groomed trails or suggested routes, while approximately 11,000 are packed‐trail cross‐country skiers and snowshoers using the 10th Mountain Hut back‐country hut system (U.S.D.A. Forest Service 2015). A developed ski resort is also near the Vail Pass study area; this resort is among the top 10 largest ski resorts in Colorado. It encompasses approximately 10.1 km^2^ of skiable area and has 23 chairlifts (USDA Forest Service National Visitor Use Monitoring Results, 2016). In the San Juan study area, recreation is primarily dispersed back‐country ski and snowboard use, with some motorized recreation concentrated primarily in high‐elevation areas. The developed ski resort we focused on in the San Juan study area has 8.1 km^2^ of skiable area and 18 chairlifts (telluride.com, 2017).

### 2.2 Quantifying movements of winter recreationists

To provide a spatially and temporally detailed sample of winter recreation intensity, we stationed technicians at parking areas and trailheads during winters of 2010–2013 to distribute small (5 × 8 cm) GPS units to be worn around the upper arm or affixed to a back‐pack (Qstarz International Co., Ltd., model BT‐Q1300, Position accuracy <10 m). Recreationists were informed that participation was voluntary and no personally identifiable information was collected, and offered a map of their day's movement as incentive to carry the GPS unit; response from recreationists was positive, with an acceptance rate of approximately 90% (Miller, 2016). We recorded four types of recreation activity (snowmobile‐assisted ski [hereafter hybrid], snowmobile, back‐country ski or snowboard, and packed trail cross‐country ski or snowshoe [hereafter packed‐trail ski]). Only one GPS unit was given to groups with multiple people to ensure independence among recreation tracks, although some people and/or groups may have been sampled more than once during the course of a winter, or across winters. Locations of recreationists were recorded at 5‐s intervals. If GPS units remained stationary, further locations were not collected until the device detected movement. Detailed descriptions of our methods to quantify movements of recreationists can be found in Miller, Vaske, Squires, Olson, and Roberts (2017) and Olson et al. (2017).

To quantify recreation intensity, we converted GPS point locations into density rasters using the Point Density tool in ArcGIS (ESRI, Redlands, CA, USA). To determine the best scale at which to look for lynx response to recreation, we considered GPS point densities in a circular neighborhood at radii of 30 m, 100 m, 500 m, and 1 km, chosen to reflect arbitrary distances at which lynx could reasonably be expected to respond to the sight and sound of recreationists. Upon examining the distribution of the data, only the 1 km scale had enough nonzero values to allow accurate estimation of regression parameters for all lynx (Kutner et al., 2005). We therefore considered only the 1 km scale for all analyses, and our conclusions are relevant to how lynx perceive recreation within 1 km distances. Density rasters were calculated separately for each recreation type and year (2010–2013). To ensure that lynx response was temporally coincident with recreation, we matched year of recreation intensity with year of lynx data collection; we did not attempt to temporally match recreation and lynx movement at any finer resolution than year due to limitations in sample size when lynx and recreation were paired by day or week. Thus, our analysis is prefaced on the assumption that lynx response is to seasonal intensity of recreation, rather than to direct presence of recreationists.

We also deployed infrared and magnetic trail counters at recreation portals and trail crossings throughout the study area to provide an index of recreation intensity on lynx home ranges independent of GPS tracks. Counters were in place between January and March, infrared counters affixed to trees approximately 1.5 m above the snow, and magnetic counters buried beneath the snow in trail center. Counter data was summed across the entire season and divided by the number of days each counter was deployed to provide an index of use measured as hits/day for each counter.

### 2.3 Lynx data collection

We trapped lynx in areas of high recreation or proximity to ski resorts where previous survey work indicated they were present. Lynx were captured using a specially designed box trap (Kolbe, Squires, & Parker, 2003) to minimize probability of injury. Traps were checked daily and animals were handled in accordance with International Animal Care and Use Committee (IACUC) permit AUP‐062‐13MHWB‐122013. Adult (≥3 years) lynx were fitted with Sirtrack satellite store on board GPS collars (210–230 g) with conventional VHF radio transmitters and a drop‐off mechanism. Collars were programmed to obtain a GPS location every 20 min, 24 hr per day in 2010, 2012, and 2013 and every 30 min, 24 hr per day, every other day in 2011. We considered the potential for scale dependency issues between collars with different fix‐rates (i.e., 20 min vs 30 min; Pépin, Adrados, Mann, & Janeau, 2004); however, movement rate (step‐length [km]/step duration [hr]) between collars with different duty cycles were similar (mean rate 30‐min duty cycle = 0.33 km/hr, 95% CI = −0.24–0.91 km/hr, *n* = 3 lynx‐years; 20‐min duty cycle = 0.39 km/hr, 95% CI = 0.12–0.66, *n* = 17 lynx‐years), and thus we felt confident in treating all collars similarly for analysis. Average fix‐rate across collars was 84%; collars were programmed to automatically drop off after June 1st.

We focused on lynx movement ecology (Nathan et al., 2008) collected during peak winter recreation to maximize the potential to measure responses to disturbance (January–March). We evaluated movements within 95% minimum convex polygon (MCPs) use areas for each lynx and excluded movements outside of these areas since animals performing exploratory movements may differ in behavior from those on stationary home ranges (Abrahms et al., 2017; Pépin, Adrados, Janeau, Joachim, & Mann, 2008). To split lynx movement into relevant behavior categories, we categorized lynx GPS locations as “active” or “stationary” using parameters determined from five stationary GPS collars (3,464 GPS locations, mean = 693 pts/collar, *SD* = 528) deployed under field conditions. Based on these stationary collars we calculated step length (straight line distance between two successive GPS points) and turn angles (relative turn angle between the vector from points *t* and *t*−1 and the vector from points *t* and *t* + 1) for stationary GPS points. We then used this distribution of distances and turn angles to determine threshold values to distinguish active from stationary states for collared lynx. We categorized lynx locations as “stationary” if GPS points were ≤27.02 m from the previous point (70th percentile) or had turn angles between 174^o^ and 180^o^ (90th percentile; Hurford, 2009).

### 2.4 Do lynx change movement behavior based on intensity of winter recreation?

To determine the impact of recreation intensity on lynx movement behavior, we modeled the response of lynx to a combination of environmental and recreation covariates, which were averaged separately for day and night periods. We first summarized lynx movement into temporal day (~0800:1700 hr) and night (~1700:0700 hr) periods using the “sunriset” command in R package “maptools”, which calculates the changing actual sunrise and sunset times for each day based on a given geographic location (Bivand & Lewin‐Koh, 2016). We considered two movement metrics as response variables for this analysis: movement rate (distance traveled [km] per unit time [hr] of only “active” points averaged across temporal period) and movement tortuosity (straight line distance from first to last point in a temporal period divided by summed distance between all points in a period; Benhamou, 2004).

Environmental predictor variables included proportion forest/nonforest, a binary variable based on landcover categories from the National Land Cover Database (NLCD; Homer et al., 2015), averaged across all locations in a temporal period (mean: 0.9, range: 0.1–1.0), canopy cover (percent per pixel tree canopy density, mean: 44.8%, range: 14.0%–67.4%, NLCD; Homer et al., 2015), and Euclidian distance to forest edge (shortest straight line distance from a lynx GPS point to an NLCD forest landcover category, mean: 141.8 m, range: 7.4–711.9 m). Recreation variables included 1 km recreation intensity for the four types of recreation (hybrid, backcountry ski, snowmobile, packed‐trail ski), and indicator variables for weekday/weekend, day/night, study area (San Juan/Vail), and sex. All pairwise correlations between predictor variables were <0.60, and variance inflation factor was <2.0, indicating no multi‐collinearity.

As a first step in the model‐building process, we considered three vegetation‐only models (i.e., single‐variable models using environmental covariates listed above) fit to each of the two response variables to control for the influence of habitat on lynx behavior. We selected the best performing vegetation model for each response variable and carried this base habitat model into the second step where we considered 10 candidate models that we hypothesized would test the influence of winter recreation intensity on lynx behavioral response (Supporting Information Table S1).

We used a mixed‐effects linear regression model for movement rate and tortuosity, with Lynx ID as a random intercept to control for the nonindependent nature of GPS points within a single lynx (Gillies et al., 2006). We considered the inclusion of study year as an additional random intercept to account for differences between years, but, as the majority of lynx only occurred in the dataset for a single year, the addition of this parameter did not noticeably effect model estimates, and thus we omitted it for model simplicity. We standardized $\left(x_{i}-\overset{¯}{x}/SD\right)$ all continuous covariates for ease of model fitting and interpretation. We ranked models using AIC_c_ (Akaike, 1974) and considered the best performing to have the lowest AIC_c_. We evaluated model fit using Q‐Q plots and scatterplots of fitted values versus residuals to verify the linear model assumptions of normality and homoscedasticity (Kutner et al., 2005), and calculated marginal *r* ^2^ to assess the variability explained by the fixed effects of the top‐performing model (Barton, 2015). All models were fitted using the lme4 package in R (Bates et al., 2015).

### 2.5 Do lynx avoid high intensity dispersed recreation?

To test whether lynx spatially avoided recreation within home ranges, we compared measures of recreation at lynx GPS points to a sample of random locations within a lynx's MCP home range (i.e., a third‐order used‐available resource selection function (RSF) design; Johnson, 1980; Manly et al., 2002). We generated random locations at a ratio of 1 use to 2 random, determined 1 km recreation intensity and percent canopy cover at all locations, and assigned a random “hour” value to each random point to allow temporal comparisons. Additionally, since much of the dispersed recreation on our study areas took place on groomed or user‐established trails (Miller, 2016; Olson et al., 2017), we hypothesized that lynx might respond more strongly to recreation when near to these high‐use areas. Since many trails are user‐established, and therefore no spatial data exists for them, we created trail features for each recreation type (hybrid, back‐country ski, snowmobile, and packed‐trail ski) from the high‐intensity areas (>25th percentile) delineated by a 100 m point density recreation raster. We measured the distance from each lynx GPS point and random location to the nearest trail of each recreation type. Using this distance to trail value, we created a binary variable for whether a point was near or far from a trail using thresholds of 250 m, 500 m, and 1 km. We also considered the possibility that influence from a trail would attenuate nonlinearly with distance, and thus created a decay function (e^−*α*/distance^) where α was a constant equal to 50, 100, 250, 500, or 2,500 (Carpenter, Aldridge, & Boyce, 2010; Lesmerises, Johnson, & St‐Laurent, 2016). We tested each scale of both covariates in univariate models, and kept the binary or decay variable that had the lowest AIC_c_ for each recreation type to represent response to trails in candidate models.

We then constructed a set of 10 GLMM logistic regression (Gillies et al., 2006) candidate models per recreation type to test lynx spatial response to dispersed recreation intensity and high‐intensity trails (Supporting Information Table S2). We considered univariate, additive, and interactive effects of canopy cover, to test whether canopy cover influenced lynx selection or avoidance of recreation, since lynx are closely tied to dense forest cover (Holbrook, Squires, Olson, DeCesare, & Lawrence, 2017; Squires, Decesare, Kolbe, & Ruggiero, 2010). We considered an interaction between recreation metrics and study area to test lynx response to differences in the quality of recreation between study areas (see Methods), and an interaction between recreation and time of day (day ~0800:1700 hr, night ~1700:0700 hr) to determine whether lynx exhibited a temporal response to recreation intensity or recreation trails. We included Lynx ID as a random intercept to control for repeated GPS locations among lynx, and weighted observations to create an equal contribution between the unbalanced used to available samples (Gillies et al., 2006). We standardized covariates as above and used AIC_c_ to select the best performing model for each recreation type. We assessed model fit using fivefold cross‐validation of the best model for each recreation type (Hastie, Tibshirani, & Friedman, 2001). We split the data into five equal partitions, re‐fit models on four partitions and predicted the outcome on the withheld partition. Since our response variable was binary, we calculated the area under the curve (AUC) of the receiver operating characteristic for each fold of the data; this provides a metric ranging between random predictive ability (0.5) and perfect model prediction (1.0; Boyce, Vernier, Nielsen, & Schmiegelow, 2002).

Additionally, to test specifically whether lynx adjusted their activity in response to temporal differences in recreation, we examined the relationship between recreation intensity and activity state (moving or stationary) at lynx GPS points. We used a GLMM to predict whether a point was active or stationary in response to the interaction of 1 km recreation intensity and time of day (day ~0800:1700 hr, night ~1700:0700 hr), with lynx ID included as a random intercept and a binomial link (Gillies et al., 2006). We also evaluated whether temporal response differed based on study area by creating a combined variable of study area and temporal period (San Juan day, San Juan night, Vail day, Vail night), to allow separate estimation of a temporal response at each study area. We performed separate models for each recreation type (Supporting Information Table S3), standardized recreation intensity metrics for ease of model fitting, and fitted models using the lme4 package in R (Bates et al., 2015). We cross‐validated top‐performing models as above to assess model fit (Hastie et al., 2001).

Finally, we tested whether lynx change their response to recreation depending on how much of it is available (i.e., a functional response; Mysterud & Ims, 1998). Functional responses can help reveal response thresholds which may be difficult to detect from individual selection or avoidance (Mysterud & Ims, 1998). For each individual, we calculated mean 1 km recreation intensity at used versus available locations within home ranges (Holbrook et al., 2017; Laforge, Brook, van Beest, Bayne, & McLoughlin, 2015). We then tested for functional responses by modeling use as a function of availability for each recreation type. We considered linear and quadratic models, and used likelihood‐ratio tests to determine which model form best fit the data (Kutner et al., 2005). A functional response, indicating disproportional changes in use in response to availability, was supported when the quadratic form was best‐fitting, or when the slope of the linear response did not equal 1 (Holbrook et al., 2017; Mysterud & Ims, 1998). In addition, for each lynx, we calculated the selection ratio (mean use/mean available) for each type of recreation (Manly et al., 2002; Mysterud & Ims, 1998). Selection ratios below 1 indicate avoidance (use less than availability), while selection ratios above 1 indicate selection (use greater than availability). We plotted selection ratio for each individual against average recreation availability in the home range to better visualize differences in patterns of lynx selection for recreation with changing availability.

### 2.6 Do lynx avoid developed recreation?

Two developed ski areas were adjacent to lynx home ranges in our study areas. As an initial test to determine the impact that such permanent, spatially concentrated centers of recreation activity had on Canada lynx space use, we performed a simple bootstrap comparison to test whether individual lynx entered ski areas less than random expectation (Manly, 2007; Manly et al., 2002). We sampled random locations distributed across each lynx's 95% MCP home range (sample size equal to the total GPS locations collected for each lynx) 1,000 times with replacement; at each iteration, we recorded the number of random locations inside the ski area boundary. We then calculated the 2.5 and 97.5 percentile from the bootstrap distribution for each lynx, and compared that to the actual number of GPS points inside the ski area boundary; a value outside either of these percentiles indicated avoidance or preference, respectively (Manly, 2007).

Next, we tested whether factors associated with the intensity of human use of the ski area influenced the probability of lynx use. For this analysis, we included all lynx points collected from January to June to evaluate if lynx use changed with decreased winter recreation. We modeled whether a lynx GPS point was in or out of the ski area boundary as a function of day of the week (weekend or weekday), since weekday use has been shown to be less intense than weekend, as well as time of day, since daylight hours receive more use than dark (Olson et al., 2017). We also considered month as a continuous variable, from February to June, since use of the ski area should decrease with later months as snowpack decreases. Finally, we included canopy cover at each GPS location to control for differences in vegetation inside and outside the ski area boundary. We used GLMMs with individual lynx ID as a random effect (Gillies et al., 2006).

We considered a candidate model set of 11 models to evaluate lynx use of developed ski areas (Supporting Information Table S4). All models contained a base structure of canopy cover to account for habitat differences inside or outside of the ski area boundary. We evaluated additive combinations of month, time of day, and day of the week, as well as interactions that we hypothesized might impact the likelihood of lynx use of the ski area (Supporting Information Table S4). We fit models using the lme4 package in R (Bates et al., 2015) and ranked models according to AIC_c_. We validated the best‐performing model using fivefold cross‐validation, as detailed above (Hastie et al., 2001).

## 3 RESULTS

We captured 18 individual lynx (9 males, 9 females) from 2010 to 2013, with four individuals captured in two successive years, for a total of 22 yearly lynx home ranges. We collected a total of 34,405 GPS points (average: 1,720/lynx, *SD*: 1100) from January to March. Lynx moved a mean of 8.0 km per day (*SD*: 4.9 km), at an average rate of 0.63 km/hr (*SD*: 0.27 km/hr).

**Figure A1:**
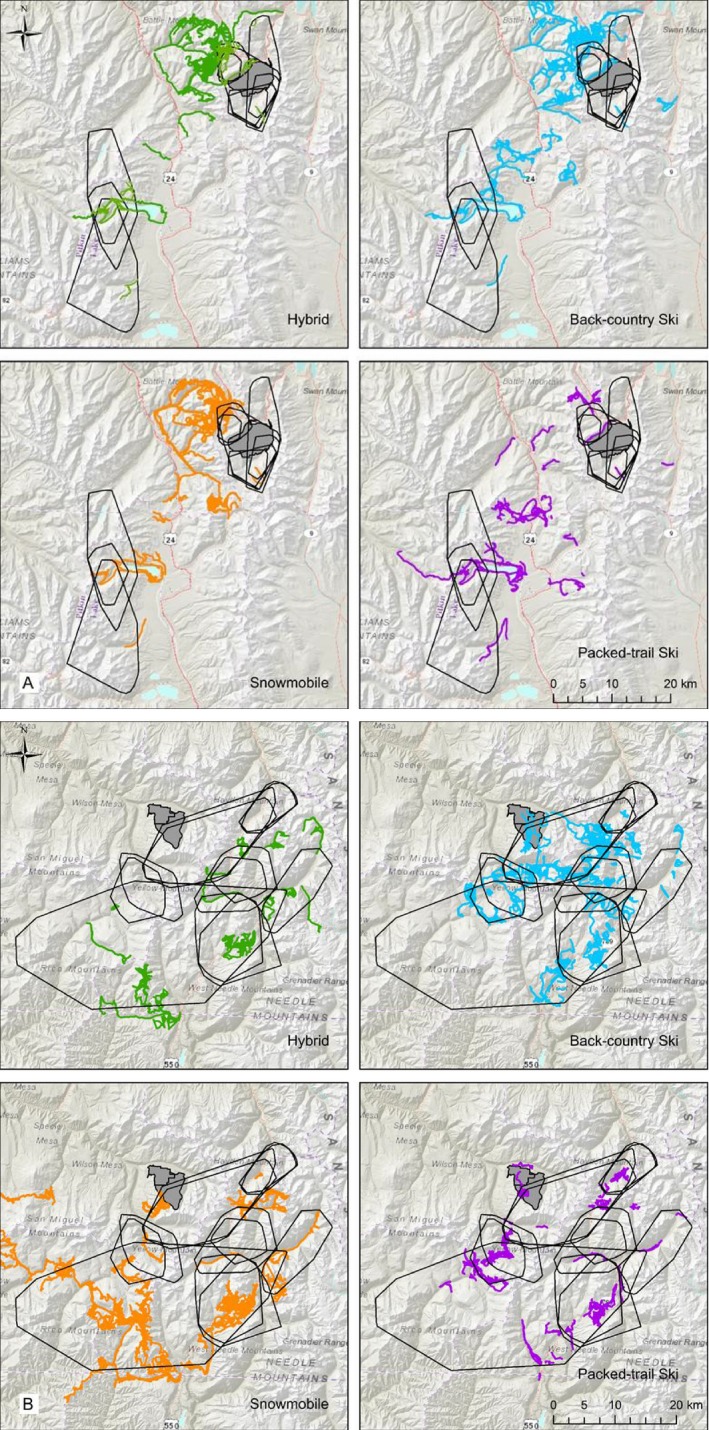
Maps showing the location of yearly Canada lynx 95% minimum convex polygon home ranges (black lines), overlapped with recreation tracks (colored lines; green = snowmobile‐assisted hybrid skiing, blue = back‐country ski, orange = snowmobile, purple = packed‐trail ski) created by recreationists carrying handheld GPS devices in western Colorado, USA, 2010–2013. Gray polygon indicates a ski area boundary. Panel A shows lynx home ranges at the Vail study area, panel B shows the San Juan study area. Background image credit: Esri, DeLorme, USGS, NPS

We collected a total of 2,839 tracks from recreationists (2010: *n* = 350, 2011: *n* = 1015, 2012: *n* = 651, and 2013: *n* = 823). Although all lynx were captured in areas used by winter recreationists, recreation intensity was highly variable across lynx (Appendix A: Figure A1). Hybrid recreation occurred on 13 yearly lynx MCP home ranges, snowmobile on 17, and backcountry ski and packed‐trail ski on 19. Recreation availability also differed between the two study areas; mean number of unique GPS tracks recorded on lynx home ranges was: hybrid) 27.8 Vail (*SD* = 55.4), 3.0 San Juan (*SD* = 4.5); back‐country ski) 11.8 Vail (*SD* = 10.4), 71.2 San Juan (*SD* = 51.3); snowmobile) 32.6 Vail (*SD* = 49.7), 20.7 San Juan (*SD* = 28.5); packed‐trail ski) 7.9 Vail (*SD* = 11.2), 66.4 San Juan (*SD* = 51.9). The mean length of all recreation tracks combined within home ranges was 10.9 km/km^2^ (*SD* = 24.0) for Vail and 9.7 km/km^2^ (*SD* = 6.7) for San Juan (Appendix A: Table A1). Trail counters had a mean of 35.9 hits/day (*SD* = 26.8) for Vail and 18.4 hits/day (*SD* = 10.8) for San Juan (Appendix A: Table A1).

### 3.1 Do lynx change movement behavior based on intensity of winter recreation?

Both lynx movement rate and movement tortuosity were best modeled by a combination of recreation and environmental variables (Supporting Information Table S1). Lynx movement rates were a function of proportion forest, recreation intensity, and sex (Table 1), with a marginal *r* ^2^ of 0.12. Lynx slowed their movement rate in the presence of greater snowmobile and back‐country ski activity. For example, predicted female lynx movement rate was 0.47 km/hr (*SD* = 0.03) with no recreation within 1 km, 0.22 km/hr (*SD* = 0.07) at maximum observed back‐country ski intensity (equivalent to approximately 66 recreation tracks/km^2^ in a season), and 0.25 km/hr (*SD* = 0.11) at maximum snowmobile intensity (approximately 188 tracks/km^2^ in a season). Conversely, at high hybrid and packed‐trail ski intensities, lynx generally moved faster, although the confidence interval for packed‐trail ski slightly overlapped zero, indicating that lynx movement rate was not as strongly related to packed‐trail ski intensity. Modeled movement rate was 0.47 km/hr (*SD* = 0.03) with no recreation within 1 km, 0.92 km/hr (*SD* = 0.21) at maximum observed hybrid intensity (equivalent to approximately 232 tracks/km^2^ in a season), and 0.56 km/hr *(SD* = 0.08) at maximum observed packed‐trail ski intensity (equivalent to approximately 115 tracks/km^2^ in a season). In addition, female lynx moved more slowly than males, and movement rate was slower with greater proportion forest.

**Table 1:** Coefficients (*β*) and confidence intervals (95% CI) for top‐performing models of Canada lynx movement rate and movement tortuosity in response to recreation intensity at a 1 km scale and other covariates in western Colorado, USA, 2010–2013

Covariate	*β*	Lower CI	Upper CI
Movement rate			
** Hybrid1k**	0.02	0.00	0.05
** Back‐country ski1k**	−0.04	−0.06	−0.02
** Snowmobile1k**	−0.02	−0.05	0.00
** **Packed‐trail ski1k	0.01	−0.01	0.03
** Proportion forest**	−0.03	−0.05	−0.01
** Sex**	0.22	0.15	0.29
Movement Tortuosity			
** **Hybrid1k	−0.01	−0.03	0.00
** **Back‐country ski1k	0.00	−0.02	0.02
** **Snowmobile1k	0.00	−0.01	0.02
** **Packed‐trail ski1k	−0.01	−0.02	0.01
** Proportion forest**	−0.02	−0.04	−0.01
** Night**	−0.05	−0.07	−0.02

Covariates whose 95% CI did not overlap 0 are bolded.

The top performing model for tortuosity included recreation, proportion forest, and time of day (Table 1), with a marginal *r* ^2^ of 0.02, indicating that this model explained little of the variation in tortuosity. Based on beta coefficient confidence intervals, recreation intensity was not an important predictor of tortuosity; lynx movement was more tortuous with greater proportion forest and during the night.

### 3.2 Do lynx avoid high intensity dispersed recreation?

Lynx space use within home ranges was better predicted with the addition of dispersed recreation covariates than with canopy cover alone; recreation intensity was most predictive for motorized recreation, and distance to trails most predictive for nonmotorized recreation (Supporting Information Table S2). The interaction with time of day was not ranked highly for any type of recreation, indicating little support for the hypothesis that lynx temporally adjusted their space use in response to recreation (Supporting Information Table S2). Cross‐validation of each model indicated acceptable model fit (hybrid: AUC = 0.74, *SD* = 0.01, back‐country ski: AUC = 0.74, *SD* = 0.01, snowmobile: AUC = 0.74, *SD* = 0.01, and packed‐trail ski: AUC = 0.75, *SD* = 0.01).

**Figure 2:**
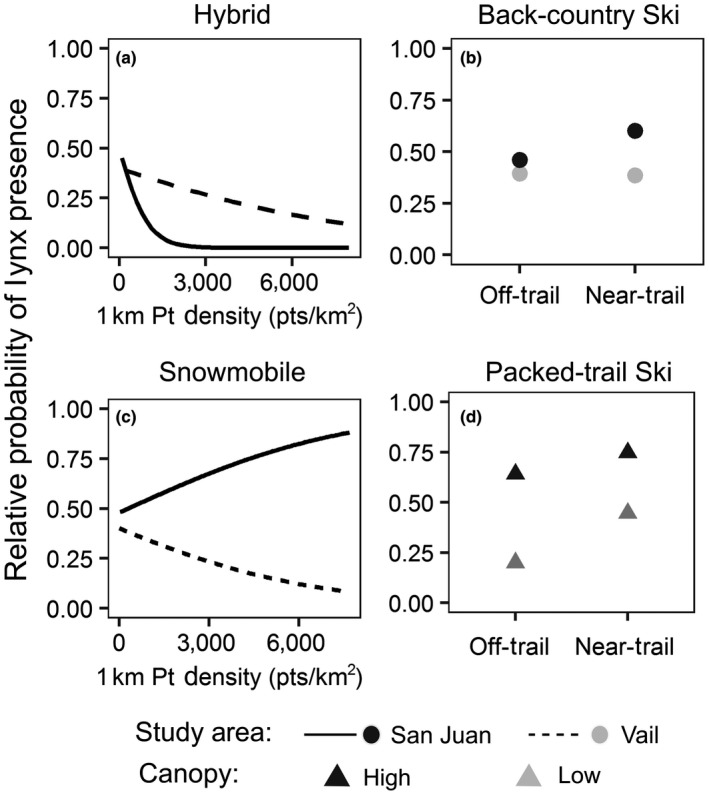
Predicted relative probability of Canada lynx presence in response to four recreation types: snowmobile‐assisted hybrid ski, back‐country ski, snowmobile, and packed‐trail ski, in western Colorado, USA, 2010–2013. Lynx (a) avoided greater hybrid intensity, (b) were more likely to be present near (<250 m) back‐country ski trails in the San Juan study area, (c) avoided greater snowmobile intensity in the Vail study area but not the San Juan study area, and (d) were more likely to be present near (<500 m) packed‐trail ski trails, particularly in areas with low canopy cover. Predictions were generated for each recreation type from multivariate general linear mixed‐effects models by holding all other covariates at their mean (see Table 2)

Lynx in the Vail study area avoided areas with greater snowmobile recreation intensity, while lynx in the San Juan study area were more likely to use them (Figure 2; Table 2); greater hybrid recreation intensity was consistently avoided at both study areas, although the effect was stronger in the San Juan than in Vail. Lynx selected areas within 250 m of back‐country ski trails and within 500 m of packed‐trail ski trails; an interaction with study area was supported for back‐country skiing, indicating that the selection for areas near to trails was stronger in the San Juan study area, while an interaction with canopy cover was selected for packed‐trail skiing, indicating that lynx were less affected by trail proximity in areas with greater canopy cover, and more likely to be influenced by trails when cover was low (Figure 2; Table 2). In addition, for all forms of recreation, the predicted probability of lynx presence was always greater with greater canopy cover; this effect tended to be stronger than that of recreation (Table 2).

**Table 2:** Coefficients (*β*) and confidence intervals (95% CI) from top‐performing models of Canada lynx use versus availability in response to recreation intensity at a 1 km scale or proximity to a recreation trail, canopy cover, and study area in western Colorado, USA, 2010‐2013

Covariate	Coefficient	Lower CI	Upper CI
Hybrid			
** Hybrid Intensity1k**	−1.23	−1.48	−0.97
** Canopy Cover**	1.05	1.03	1.06
** Study Area**	−0.27	−0.43	−0.11
** Hybrid1k:Area**	1.10	0.84	1.36
** **Random Effect	Var: 0.03	*SD*: 0.17	
Back‐country Ski			
** Back‐country Ski Trail250 m**	0.57	0.53	0.62
** Canopy Cover**	1.04	1.02	1.06
** Study Area**	−0.27	−0.45	−0.09
** BC‐SkiTrail:Area**	−0.61	−0.74	−0.49
** **Random Effect	Var: 0.04	*SD*: 0.20	
Snowmobile			
** Snowmobile Intensity1k**	0.19	0.17	0.21
** Canopy Cover**	1.06	1.04	1.08
** Study Area**	−0.42	−0.57	−0.27
** Snowmobile1k:Area**	−0.38	−0.42	−0.34
** **Random Effect	Var: 0.03	*SD*: 0.16	
Packed‐trail Ski			
** Packed‐trail Ski Trail500 m**	0.84	0.80	0.89
** Canopy Cover**	1.10	1.08	1.12
** PT‐SkiTrail:Canopy**	−0.37	−0.42	−0.32
** **Random Effect	Var: 0.08	*SD*: 0.28	

Covariates whose 95% CI did not overlap 0 are bolded.

**Figure 3:**
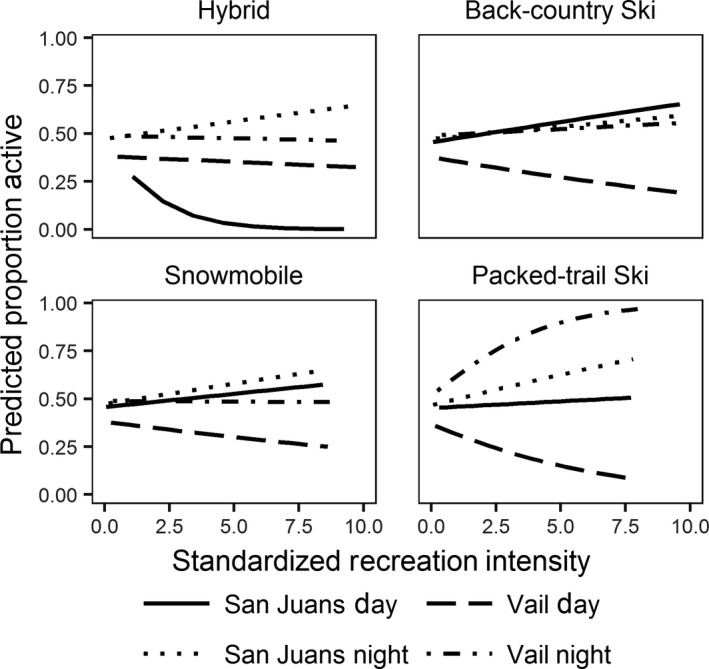
Predicted differences in temporal lynx activity patterns in response to four types of recreation intensity (snowmobile‐assisted hybrid ski, back‐country ski, snowmobile, and packed‐trail ski) in western Colorado, USA, 2010–2013. Line type represents day or night at the Vail or San Juan study areas. Proportion of time spent active was similar at low recreation intensities, but diverged as recreation intensity increased, with activity primarily lowest during the day at the Vail study area

Lynx temporal activity in response to recreation intensity was best modeled when allowed to vary with study area and time of day (Supporting Information Table S3). In general, at areas with no recreation tracks within 1 km (i.e., recreation intensity = 0), the proportion of time that lynx spent active was fairly similar during the day and night and across study areas (Figure 3). As recreation intensity of any type increased, however, lynx activity decreased during the day and increased at night in the Vail study area. Conversely, lynx in the San Juan study area were less active during the day than night in areas with greater packed‐trail ski and hybrid ski, but not back‐country ski or snowmobile (Figure 3; Supporting Information Table S5). Cross‐validation of selected models, however, found relatively poor model predictive performance (hybrid: AUC = 0.56, *SD* = 0.01, back‐country ski: AUC = 0.57, *SD* = 0.01, snowmobile: AUC = 0.57, *SD* = 0.01, packed‐trail ski: AUC = 0.57, *SD* = 0.01), indicating that though lynx temporal activity was influenced by recreation intensity and study area, much of the variation in temporal activity remained unexplained.

**Figure 4:**
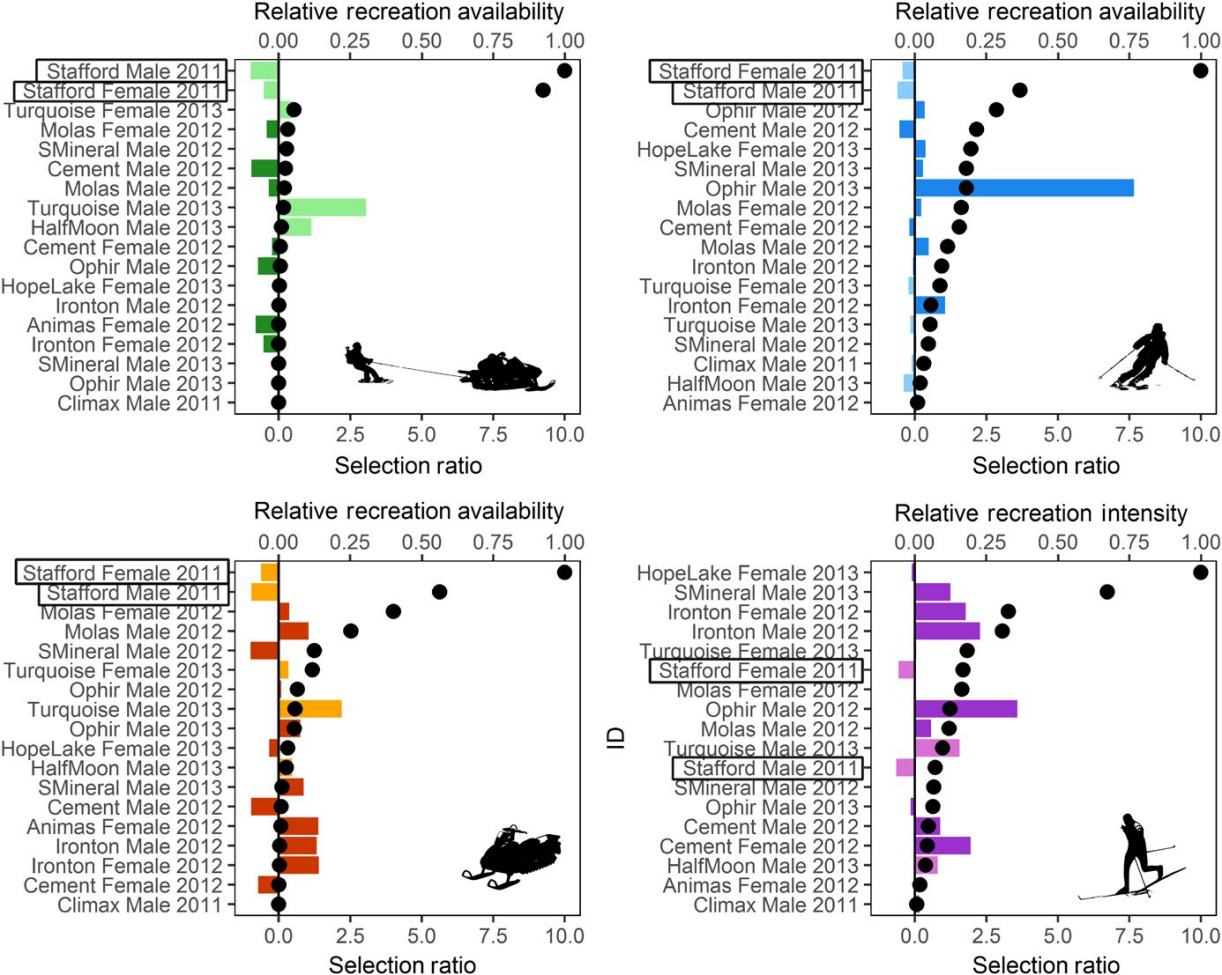
Individual selection ratios (mean use/mean availability; colored bars) compared to mean relative recreation availability (black dots) for each recreation type (a) snowmobile‐assisted hybrid ski, (b) back‐country ski, (c) snowmobile, (d) packed‐trail ski. For ease of interpretation, selection ratio‐1 is shown, so that avoidance is below 0, selection above 0, and availability values are relative (each divided by the maximum availability value) to force the range between 0 and 1. Lynx in each study area are indicated by dark bars (San Juan) or light bars (Vail). Stafford Female and Stafford Male (names outlined in boxes), from the Vail study area, are the only lynx to consistently avoid all types of recreation, and have the highest availability for 3 out of the 4 recreation types

We found support for functional responses to hybrid (Likelihood Ratio Test *p *=* *0.01, *r* ^2^ = 0.78), snowmobile (LRT *p *=* *0.05, *r* ^2^ = 0.49), and packed‐trail ski recreation (LRT *p *=* *0.01, *r* ^2^ = 0.83), with mean use best modeled by a quadratic response to mean availability; back‐country ski intensity did not support a functional response (LRT *p *=* *0.27, *β*_0_: 219.70, 95% CI: −164.57–603.96, *β*_1_: 0.60, 95% CI: −0.13–1.32, *r* ^2^ = 0.14). Lynx used areas with hybrid and snowmobile recreation in proportion to availability when recreation intensity was low; however, as recreation intensity increased, lynx use appeared to decrease. For packed‐trail ski intensity, lynx use appeared to be proportional to availability at low and high intensity, but greater than availability at moderate intensities, while use of areas with back‐country skiing were proportional to availability. Among our sampled lynx, most had relatively low recreation availability, however, so that the few individuals with high availabilities may have driven results. The plotted selection ratios (Figure 4), which allowed better visualization of selection across the range of recreation intensity, demonstrated no consistent pattern of selection or avoidance at low availabilities, but showed that the only two lynx with consistent avoidance also had the highest recreation availabilities for three out of four types of recreation (Figure 4, highlighted boxes).

### 3.3 Do lynx avoid developed recreation?

**Figure 5:**
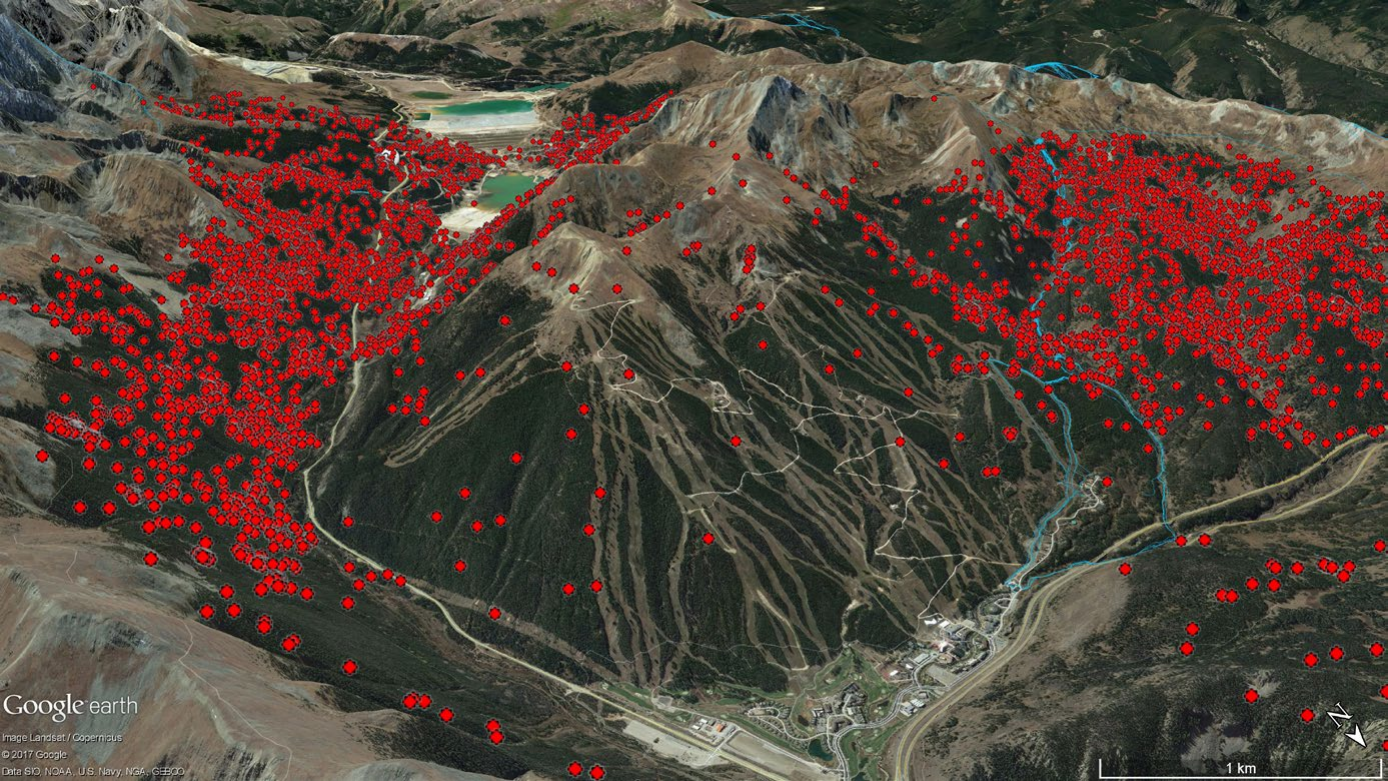
The spatial distribution of Canada lynx GPS points (red dots) around the ski resort in the Vail Pass study area, in western Colorado, USA, 2010–2013; lynx locations indicate avoidance of this heavily recreated area. Individual ski runs are the light‐colored lines in the center of the picture, while the resort infrastructure is toward the bottom right. Lower intensity dispersed skiing (blue lines) along a groomed trail to a back‐country hut is shown to the right of the ski area; lynx did not exhibit spatial avoidance of this type of use

We captured lynx near two ski resorts, one near the Vail Pass Winter Recreation area and the other in the San Juan Mountains. Five unique lynx, two captured in successive years, had home ranges adjacent to the ski area near the Vail Pass study area, while one unique lynx, captured in two successive years, was adjacent in the San Juan study area. On average, lynx yearly 95% MCP home ranges overlapped ski areas 15.8% (*SD*: 8.9%), ranging from 3.9% to 27.4% (*n* = 951 total lynx locations inside ski area boundaries, *n* = 22,524 lynx locations outside ski area boundaries). Of these nine lynx‐years near developed recreation, 8 had fewer locations inside ski area boundaries than expected, indicating avoidance, while one did not differ from random (Table 3; Figure 5).

**Table 3:** Summary of the number of Canada lynx GPS locations (N) inside two ski resort boundaries in western Colorado, USA, compared to the bootstrapped 95% confidence values (CI) for each lynx

Lynx ID	Study area	Year	*N*	Lower CI	Upper CI
**Breckenridge Female 2010**	Vail	2010	78	466	544
**Breckenridge Female 2011**	Vail	2011	116	322	392
**Climax Female 2010**	Vail	2010	11	87	126
**Climax Male 2011**	Vail	2011	29	141	182
**Stafford Female 2010**	Vail	2010	92	243	295
Stafford Female 2011	Vail	2011	366	329	396
**Stafford Male 2011**	Vail	2011	204	618	700
**Ophir Male 2012**	San Juan	2012	0	87	127
**Ophir Male 2013**	San Juan	2013	55	134	181

Lynx that avoided ski areas (actual GPS points less than the lower 2.5% bootstrapped value) are bolded.

The top supported model of lynx‐use in developed ski areas included all covariates and an interaction between month and day of the week (Supporting Information Table S4), and fivefold cross‐validation indicated the model had adequate predictive ability (mean AUC = 0.77, *SD* = 0.02). Lynx were more likely to enter the ski area boundary during night than day (Table 4). Additionally, an interaction between month and day of week was strongly supported: on weekends, lynx use of ski areas increased with month as months became warmer and winter recreation declined, so that predicted use on June weekends was 4.7 times that of February weekends. During weekdays the effect of month was less pronounced, with predicted June use only 1.1 times greater than predicted February use. Canopy cover was weakly lower at lynx locations inside the ski area than outside, although the model with only canopy cover ranked second to last among the candidate models, indicating that habitat alone was a poor predictor of lynx use of the ski area (Supporting Information Table S4).

**Table 4:** Coefficients (*β*) and confidence intervals (95% CI) from the top‐performing model of lynx use of ski resorts in western Colorado, USA, 2010–2013

	Coefficient	*SE*	Lower CI	Upper CI
**Intercept**	−4.20	0.55	−5.28	−3.12
**Month**	0.18	0.03	0.11	0.24
**Weekend**	−2.00	0.31	−2.61	−1.39
**Night**	0.14	0.07s	0.01	0.28
Canopy Cover	−0.07	0.03	−0.14	0.00
**Month**:**Weekend**	0.38	0.07	0.25	0.52

Covariates whose 95% CI did not overlap 0 are bolded.

## 4 DISCUSSION

Our results demonstrate a nuanced response of Canada lynx to winter recreation, ranging from avoidance of developed ski resorts to tolerance of nonmotorized back‐country skiing and packed‐trail skiing. Taken together, lynx spatial and behavioral responses to the gradient of recreation recorded in our study may suggest a tolerance threshold, with little disturbance from low and moderate intensity recreation but increasing disturbance when intensity exceeds a given level. For instance, evidence of temporal avoidance of recreation was most marked in the high‐intensity Vail study area. Functional responses of use versus availability also indicated little evidence of avoidance when recreation availability was low, but consistent avoidance for the two lynx with the greatest recreation availability. Lynx also consistently avoided developed ski resorts, especially at times when recreation was most intense. In areas with low and moderate recreation intensity, lynx exhibited spatial tolerance coupled with behavioral modifications that allowed lynx and dispersed recreation to co‐occur. Based on these results, it appears that dispersed winter recreation at the low to moderate intensities found in western Colorado does not provoke a strong negative response in Canada lynx, but that high‐intensity dispersed or developed recreation may provide enough of a disturbance to elicit lynx avoidance.

Animal responses to human disturbance often vary depending on the type of disturbance activity. Wild reindeer fled longer distances when disturbed by skiers than snowmobiles (Reimers, Eftestøl, & Colman, 2003), while moose exhibited short‐term disturbance from skiers (Neumann, Ericsson, & Dettki, 2010) and avoidance of areas with high snowmobile trail density (Harris et al., 2013). Lynx did not appear to exhibit a consistent response to all dispersed recreation types, although some consistent differences between motorized and nonmotorized recreation types emerged. Areas with greater nonmotorized recreation intensity (i.e., back‐country and packed‐trail ski,) were selected by lynx, while areas with greater snowmobile and hybrid recreation intensity were generally avoided. This pattern may reflect similarities between the habitat preferences of lynx and skiers, and habitat differences between lynx and motorized recreation. For instance, nonmotorized recreation is frequently located in high elevation areas, with dense canopy cover and steep slopes (Olson et al., 2017), habitat which is likely favored by forest‐dwelling Canada lynx, which prefer areas with multi‐storied forest and high horizontal cover (Holbrook et al., 2017; Squires et al., 2010). Motorized recreation such as snowmobiling, however, usually takes place on groomed trails or forest roads, which are placed in areas of open forest and gentle topography to allow safer fast travel (Olson et al., 2017), and which is not as hospitable to lynx.

Developed recreation may be more likely to have an effect on animals, given the high intensity, large infrastructure, and frequent maintenance requirements of large ski resorts (Rixen & Rolando, 2013). For example, Pacific marten (*Martes caurina*) have been shown to be negatively influenced by ski resorts through habitat fragmentation and reduced occupancy and density during the winter season (Slauson, Zielinski, & Schwartz, 2017). Lynx also appeared to be affected by developed recreation, although our sample size for this analysis was small. Lynx near developed ski resorts appeared to spatially avoid the ski area (Figure 5), and to temporally adjust their activity to avoid high traffic times when they did go near it, even after controlling for differences in vegetation between the ski area and its surroundings. Lynx avoidance of intensely used ski resorts also supports the idea of a threshold level of tolerance toward human disturbance, with lynx able to adjust their space use or behavior in the presence of most dispersed recreation, but unable to tolerate high levels of human use that occur at a resort. Developed resorts in Colorado are intensively used, and are also subject to frequent motorized trail grooming and maintenance. Combined, this may represent unacceptably high levels of human disturbance for lynx. Other species have shown similar avoidance of developed ski areas, including mountain goats (Richard & Côté, 2016), reindeer (Nellemann et al., 2010), and alpine black grouse (Patthey et al., 2008).

Behaviorally, lynx tended to move more slowly in areas with greater snowmobile and back‐country ski intensity, while their movement tortuosity remained unchanged. This behavioral change may indicate that lynx perceive a threat from human disturbance, and respond by hiding or moving more cautiously (Tablado & Jenni, 2017), but do not change their foraging behavior by either stopping completely or moving directly out of an area. While increased movement rate or flight responses are common indications of disturbance (Arlettaz et al., 2015; Reimers et al., 2003), hiding or freezing are also common behavioral responses to threats (Tablado & Jenni, 2017). Lynx may rely on their cryptic coloration to protect them from notice, thus saving themselves from a more energetically costly flight response (Stankowich & Blumstein, 2005). While lynx did not exhibit strong temporal avoidance of recreation, they adjusted the proportion of time they spent active in areas with greater recreation, particularly in the high‐intensity Vail study area, in which they were less active during the day. Rather than leave high intensity areas during the day, lynx may simply become less active and more cautious, waiting for the disturbance to decline and increasing their activity at night. Temporal avoidance is frequently observed in response to human disturbance, and has been demonstrated in predators such as coyotes (*Canis latrans*) and bobcats (*Lynx rufus*) (Reilly, Beier, & Sonderegger, 2016; Riley et al., 2003).

While we focused our study on some of the most heavily recreated landscapes in Colorado, collected during peak winter recreation to maximize the potential to measure responses to disturbance, the lack of consistent avoidance may suggest that low to moderate dispersed recreation at our study areas was not intense enough to elicit a strong population‐level response from lynx. Response to recreation can vary at the level of the individual, often depending on an individual's age, sex, reproductive status, or other factors (Lesmerises & St‐Laurent, 2017; Tablado & Jenni, 2017). The results of our functional response analysis indicate that lynx in our study varied in their selection or avoidance of recreation, with differences both between individuals in response to the same type of recreation, and within individuals given different types of recreation (Figure 4). Interestingly, the two lynx in areas with the greatest amount of recreation also demonstrated the most consistent avoidance. These lynx had the highest availability of recreation intensity for three out of the four recreation types (Figure 4), and were located in the Vail study area, which had extremely high intensity use, approximately 35,000 recreationists per winter (U.S.D.A. Forest Service, 2015), as well as a large developed ski area. Thus, recreation intensity in our study area may be low enough for the majority of lynx to ignore and spatially coexist with, but an intensity threshold may exist above which dispersed recreation cannot be tolerated by lynx. We recognize that behavior responses are not necessarily expressed in changes in population demography and adult survivorship. However, the population of lynx in Colorado has been recovering since reintroduction in 1999, and is currently estimated at 200 to 300 individuals (Martin, 2013). Our sample likely represents approximately 6%–9% of the entire lynx population in Colorado, and the majority of resident lynx at each study area, and illustrates that a sizeable portion of the population is subject to disturbance from recreation.

Carnivores are often reported to be particularly sensitive to anthropogenic disturbance because of their need for large contiguous home ranges and a tendency to draw human persecution (Carroll, Noss, & Paquet, 2001; Noss, Quigley, Hornocker, Merrill, & Paquet, 1996; Woodroffe, 2000). However, both Canada lynx and Eurasian lynx (*Lynx lynx*) have demonstrated a high degree of tolerance to human presence. For example, Eurasian lynx in Norway show a preference for low levels of human disturbance in their home ranges, but will tolerate extremely urban areas, possibly because of a correlated increase in prey availability (Bouyer et al., 2014, 2015). Similarly, Canada lynx in Riding Mountain National Park, Canada, tended to have high probabilities of occurrence in the less disturbed park interior, but highest occurrence near a town with intense winter recreation yet close proximity to highly suitable hare habitat (Montgomery, Roloff, Millspaugh, & Nylen‐Nemetchek, 2014). Lynx in our study also failed to exhibit strong behavioral avoidance from low to moderate intensity dispersed recreation, instead appearing to segregate themselves from high‐intensity motorized recreation and to adjust their temporal and movement patterns.

Given the nature of our study design, we were unable to evaluate the potential for second order (i.e., home‐range placement; Johnson, 1980) avoidance of recreation. The arrangement of home ranges to avoid recreation or human disturbance has been observed in northern mountain woodland caribou (*Rangifer tarandus caribou*) in response to human infrastructure (Polfus, Hebblewhite, & Heinemeyer, 2011), but was not found in capercaillie (*Tetrao urogallus*) in response to outdoor recreation (Coppes, Ehrlacher, Thiel, Suchant, & Braunisch, 2017) or red deer (*Cervus elaphus*) in response to recreation infrastructure (Coppes, Burghardt, Hagen, Suchant, & Braunisch, 2017). It is possible that some lynx may have already exhibited avoidance of recreation, through the selection of home ranges that do not overlap with recreation. If this is the case, the lynx that were trapped for our study may be habituated to recreation, and the continued occupancy of these territories in subsequent lynx generations may not be assured. Alternatively, use of high recreation intensity areas may be a function of limited habitat distribution in high elevation linear valleys, rather than habituation to recreation per se. Since it is possible that the lack of strong response to recreation we found represents the result of an ongoing strategy of avoidance by lynx sensitive to recreation, we recommend long‐term monitoring of lynx occupancy near heavily recreated areas to ensure that lynx are not negatively impacted by recreation (with the assumption that continued occupancy reflects a lack of detrimental demographic effects). Further, to thoroughly evaluate causal impacts of recreation to lynx in Colorado, we suggest continued research to measure demographic (and/or behavioral) responses to experimental manipulation of user access and density.

## 5 CONCLUSIONS

We evaluated a gradient of human disturbance from winter recreation, from intensively used developed ski areas to low‐intensity dispersed back‐country recreation. In keeping with this range of disturbance, we found a range of Canada lynx responses to winter recreation, from avoidance of developed ski resorts to tolerance of nonmotorized back‐country skiing and packed‐trail skiing. Lynx may tolerate low to moderate levels of dispersed winter recreation (similar to levels we sampled at the San Juan Study Area) via behavioral modifications governing activity levels, activity timing, and locations of various activities. Thus, recreation management such as trail closures, visitor limitation, etc., may convey little benefit to species conservation in areas with low to moderate levels of dispersed recreation. We found less spatial avoidance of nonmotorized recreation compared to motorized, although lynx response varied by study area, and lynx exhibited a behavioral response to both motorized and nonmotorized recreation. Our results support the conclusion that lynx, as evidenced by changes in space use and behavior, were not uniformly negatively influenced by dispersed winter recreation at the low to moderate intensities found in our study. However, lynx residing in more heavily recreated landscapes left stronger response signatures, culminating in fairly strong avoidance of the most intensely recreated landscapes, such as commercial ski areas. While behavioral changes do not necessarily reflect impacts on survival or reproductive success, if lynx conservation is an important goal in these areas, implementation of programs to alleviate recreation intensity may be considered. Alternatively, intense recreation could be administratively concentrated, leaving space for lynx conservation measures in adjacent regions.

## CONFLICT OF INTEREST

None declared.

## AUTHORS CONTRIBUTION

J. Squires and E. Roberts conceived the concepts, J. Squires, E. Roberts, J. Ivan, L. Olson, and M. Hebblewhite designed the methodology, L. Olson performed the data analyses, J. Squires, M. Hebblewhite, and J. Ivan consulted on data analyses, L Olson led the writing of the manuscript, all authors contributed critically to the drafts. All authors gave final approval for publication.

## DATA ACCESSIBILITY

Data available from the figshare repository: https://doi.org/10.6084/m9.figshare.6452807.v1

## APPENDIX 1

### 1.1

**Table A1:** Summary statistics for recreation intensity on each Canada lynx's yearly 95% minimum convex polygon home range (*n* = 22) in western Colorado, USA, 2010–2013. For each lynx's yearly home range (HR), the number of recreation tracks that we recorded of each type in home ranges is given (#), along with total length of track (km) of each type recorded in each home range, the home range size (km^2^), the density of all recreation tracks combined (linear km of recreation tracks/km^2^ home range area), and the average (Avg) and standard deviation (*SD*) of trail counter hits per day at all trail counters within each lynx's home range

Study Area	Indv ID	# Recreation tracks on HR				km of Recreation tracks on HR				HR size (km^2^)	Track density km/km^2^	Avg counter hits/day	SD counter hits/day
		Hyb	BKSki	Snmb	PTSki	Hyb	BCski	Snmb	PTSki				
Vail	Stafford Female 2011	134	27	126	2	1182	170	1079	17	31.37	78.00	53.11	47.29
Vail	Turquoise Female 2013	4	13	25	24	32	44	300	100	45.03	10.57	65.67	0.0
Vail	Stafford Female 2010	0	0	2	0	0	0	11	0	14.72	0.74	86.27	74.61
Vail	Breckenridge Female 2011	0	3	0	1	0	12	0	4	34.56	0.47	16.83	5.94
Vail	Breckenridge Female 2010	0	0	0	0	0	0	0	0	25.07	0.00	0.00	0.0
Vail	Climax Female 2010	0	0	0	0	0	0	0	0	35.09	0.00	12.81	9.82
Vail	Stafford Male 2011	132	24	123	3	494	167	435	21	92.14	12.11	24.16	28.42
Vail	Turquoise Male 2013	4	20	25	29	41	59	410	157	135.43	4.93	46.85	25.07
Vail	Half Moon Male 2013	4	14	25	18	23	41	163	66	175.41	1.67	34.38	25.33
Vail	Climax Male 2011	0	17	0	2	0	41	0	6	85.06	0.55	18.69	12.33
San Juan	Molas Female 2012	7	53	43	57	122	222	1660	215	106.77	20.79	11.21	4.86
San Juan	Cement Female 2012	1	37	0	5	4	138	0	27	48.68	3.47	4.10	2.77
San Juan	Hope Lake Female 2013	1	64	6	172	3	226	98	1118	67.36	21.45	30.25	22.07
San Juan	Animas Female 2012	1	4	1	4	1	16	40	22	79.62	1.00	8.11	1.14
San Juan	Ironton Female 2012	0	22	1	48	0	39	3	187	43.20	5.30	24.42	19.95
San Juan	South Mineral Male 2012	15	107	95	116	415	483	3269	569	663.02	7.14	11.38	9.88
San Juan	Cement Male 2012	2	102	3	8	20	328	10	40	104.52	3.80	5.87	7.57
San Juan	Molas Male 2012	7	69	43	56	122	247	1660	226	157.63	14.31	10.81	4.85
San Juan	Ophir Male 2012	2	184	29	70	35	883	508	267	175.51	9.64	29.10	19.80
San Juan	Ironton Male 2012	0	22	1	48	0	35	3	186	42.26	5.32	27.45	16.08
San Juan	South Mineral Male 2013	0	71	6	127	0	251	41	962	75.22	16.67	34.50	22.60
San Juan	Ophir Male 2013	0	127	20	86	0	658	469	279	200.30	7.02	24.15	27.32
