# Supplementary material for: Sharing the same slope: Behavioral responses of a threatened mesocarnivore to motorized and nonmotorized winter recreation
Source: Ecol Evol. 2018 Jul 30;8(16):8555–72. doi: 10.1002/ece3.4382 (PMC6144989; doi:10.1002/ece3.4382)
Supplement: Supplementary file 1 [file ECE3-8-8555-s001.docx]

Appendix Figure 1: Maps showing the location of yearly Canada lynx 95% minimum convex polygon home ranges (black lines), overlapped with recreation tracks (colored lines; green=snowmobile-assisted hybrid skiing, blue=back-country ski, orange=snowmobile, purple=packed-trail ski) created by recreationists carrying handheld GPS devices. Gray polygon indicates a ski area boundary. Panel A shows lynx home ranges at the Vail study area, panel B is the San Juan study area. Background image credit: Esri, DeLorme, USGS, NPS.


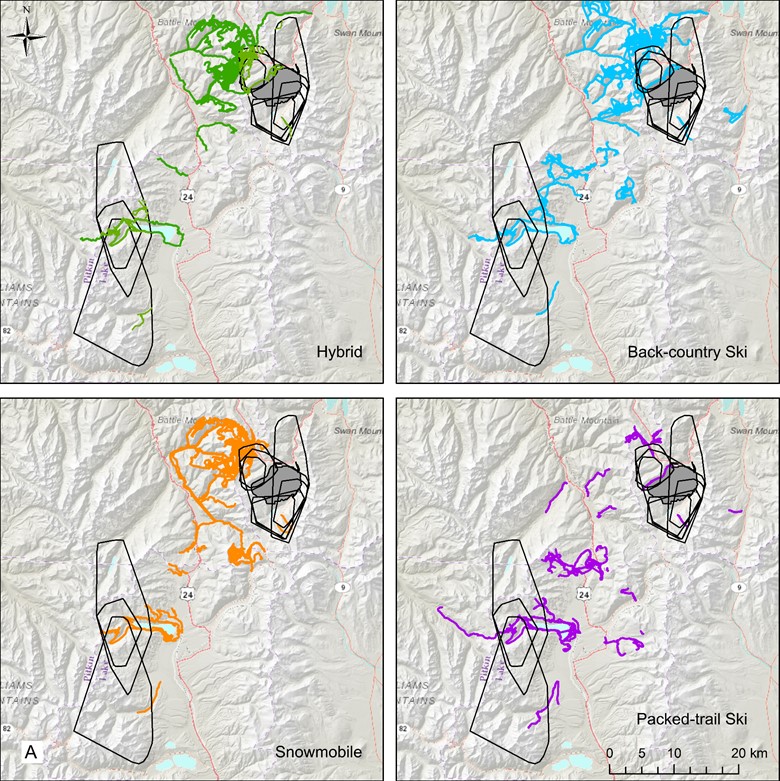


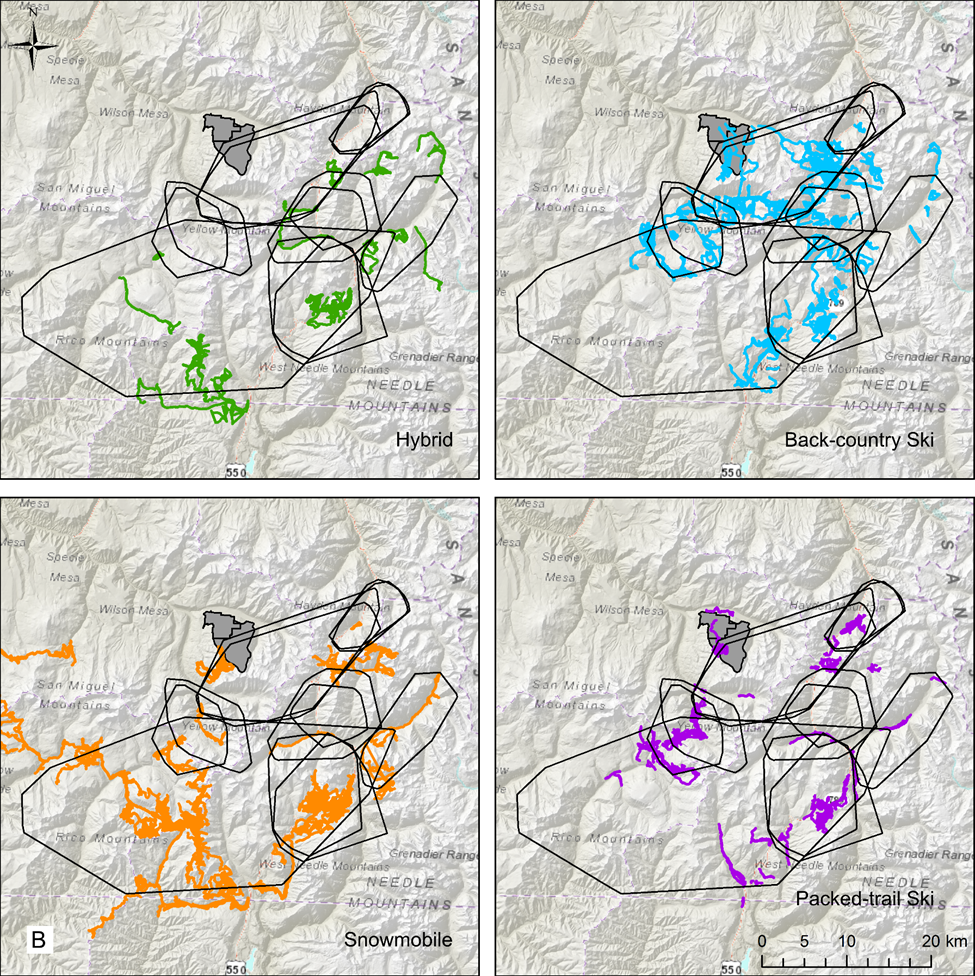


Appendix Table 1: Candidate models considered for Canada lynx movement behavior (movement speed, movement tortuosity) in western Colorado, USA, 2010-2013. Models are ranked by AIC_c_, with the best performing listed first in bold. Number of model parameters (K), difference in AIC_c_ values (ΔAIC_c_), weight of individual models (AIC_c_WT), and model log likelihood (LL) are shown. Model parameters include all 4 recreation intensity covariates (Rec; an additive formula of snowmobile, back-country ski, snowmobile-assisted hybrid ski, and packed-trail ski intensity at the 1km scale), sex of the individual (Sex), proportion of forested landcover type (Prop.Forest), weekday/weekend (DOW), day/night (TOD), and study area (Area; Vail or San Juan).

| Movement Speed | K | AIC_c_ | ΔAIC_c_ | AIC_c_Wt | LL |
| --- | --- | --- | --- | --- | --- |
| **Rec+Sex+Prop.Forest** | 9 | 591.55 | 0.00 | 1.00 | -286.70 |
| Rec*DOW+**Prop.Forest** | 13 | 603.13 | 11.58 | 0.00 | -288.41 |
| Rec+ DOW +**Prop.Forest** | 9 | 608.64 | 17.09 | 0.00 | -295.24 |
| Rec+Area+**Prop.Forest** | 9 | 612.43 | 20.88 | 0.00 | -297.14 |
| Rec+**Prop.Forest** | 8 | 613.02 | 21.47 | 0.00 | -298.45 |
| Rec***Prop.Forest** | 12 | 614.87 | 23.31 | 0.00 | -295.30 |
| Red+TOD+**Prop.Forest** | 9 | 614.95 | 23.40 | 0.00 | -298.40 |
| Rec*Area+**Prop.Forest** | 13 | 617.5 | 25.95 | 0.00 | -295.59 |
| Rec* TOD +**Prop.Forest** | 13 | 618.33 | 26.78 | 0.00 | -296.01 |
| **Prop.Forest** | 4 | 622.94 | 31.39 | 0.00 | -307.45 |
| **Null** | 3 | 633.57 | 42.01 | 0.00 | -313.77 |
|  |  |  |  |  |  |
| Tortuosity | K | AIC_c_ | ΔAIC_c_ | AIC_c_Wt | LL |
| **Rec+TOD+Prop.Forest** | 9 | -213.29 | 0.00 | 0.76 | 115.72 |
| **Rec** * **TOD** +**Prop.Forest** | 13 | -210.55 | 2.74 | 0.19 | 118.43 |
| **Rec**+Area+**Prop.Forest** | 9 | -206.01 | 7.28 | 0.02 | 112.08 |
| **Prop.Forest** | 4 | -205.98 | 7.31 | 0.01 | 107.01 |
| **Rec** +DOW+**Prop.Forest** | 9 | -205.22 | 8.07 | 0.00 | 111.68 |
| **Rec** +**Prop.Forest** | 8 | -202.02 | 11.27 | 0.00 | 109.07 |
| **Rec** *Area+**Prop.Forest** | 13 | -200.48 | 12.81 | 0.00 | 113.40 |
| **Rec** +Sex+**Prop.Forest** | 9 | -200.16 | 13.13 | 0.00 | 109.15 |
| **Rec** *DOW+**Prop.Forest** | 13 | -199.62 | 13.67 | 0.00 | 112.96 |
| Rec***Prop.Forest** | 12 | -197.46 | 15.82 | 0.00 | 110.87 |
| Null | 3 | -195.70 | 17.59 | 0.00 | 100.86 |

Appendix Table 2: Candidate models predicting Canada lynx selection (i.e., a third-order used-available resource selection function (RSF) design; Johnson, 1980; Manly et al., 2002) as a function of recreation intensity in western Colorado, USA, 2010-2013. Within recreation type, models are ranked by AIC_c_, with the best performing listed first in bold. Type of recreation modeled (Mode), number of model parameters (K), difference in AIC_c_ values (ΔAIC_c_), and model log likelihood (LL) are shown. Model parameters include recreation intensity measured at the 1km scale (Intensity1k), within 250m or not of high-intensity use trails (Trail250), within 500m or not of high-intensity use trails (Trail500), percent canopy cover (Canopy), study area (Area; Vail or San Juan), and day or night (Time).

| Mode | Model | K | AIC_c_ | ΔAIC_c_ | LL |
| --- | --- | --- | --- | --- | --- |
| Hybrid | Hyb_Intensity1k*Area + Canopy | 6 | 81556.20 | 0.00 | -40772.10 |
| Hybrid | Hyb_Intensity1k*Canopy | 5 | 81566.40 | 10.20 | -40778.20 |
| Hybrid | Hyb_Intensity1k + Canopy | 4 | 81654.50 | 104.40 | -40823.30 |
| Hybrid | Hyb_Intensity1k*Time + Canopy | 6 | 81656.79 | 116.59 | -40822.40 |
| Hybrid | Hyb_Trail1k*Area | 6 | 81660.60 | 126.65 | -40824.30 |
| Hybrid | Hyb_Trail1k*Canopy | 5 | 81672.79 | 210.00 | -40831.39 |
| Hybrid | Canopy cover | 3 | 81766.20 | 98.30 | -40880.10 |
| Hybrid | Hyb_Trail1k | 3 | 95188.30 | 100.59 | -47591.15 |
| Hybrid | Hyb_Trail1k*Time | 5 | 95190.59 | 104.40 | -47590.29 |
| Hybrid | Hyb_Intensity1k | 3 | 95213.28 | 116.59 | -47603.64 |
| Hybrid | Null | 2 | 95394.90 | 126.65 | -47695.50 |
|  |  |  |  |  |  |
| Back-country Ski | BC-Ski_Trail250*Area | 6 | 81198.67 | 0.00 | -40593.33 |
| Back-country Ski | BC-Ski_Trail250+Canopy | 4 | 81296.20 | 97.53 | -40644.10 |
| Back-country Ski | BC-Ski_Trail250*Canopy | 5 | 81296.40 | 97.73 | -40643.20 |
| Back-country Ski | BC-Ski_Intensity1k*Area + Canopy | 6 | 81363.50 | 164.83 | -40792.10 |
| Back-country Ski | BC-Ski_Intensity1k*Canopy | 5 | 81594.20 | 395.53 | -40593.33 |
| Back-country Ski | BC-Ski_Intensity1k*Time + Canopy | 6 | 81615.30 | 416.63 | -40644.10 |
| Back-country Ski | BC-Ski_Intensity1k + Canopy | 4 | 81619.00 | 420.33 | -40643.20 |
| Back-country Ski | Canopy cover | 3 | 81766.20 | 567.53 | -40675.70 |
| Back-country Ski | BC-Ski_Trail250 | 3 | 94475.08 | 13276.41 | -40792.10 |
| Back-country Ski | BC-Ski_Trail250*Time | 5 | 94476.03 | 13277.36 | -40801.65 |
| Back-country Ski | BC-Ski_Intensity1k | 3 | 94768.85 | 13570.18 | -40805.50 |
| Back-country Ski | Null | 2 | 95394.90 | 14196.23 | -40880.10 |
|  |  |  |  |  |  |
| Snowmobile | Snmb_Intensity1k*Area + Canopy | 6 | 81350.50 | 0.00 | -40669.20 |
| Snowmobile | Snmb_Trail1k*Canopy | 5 | 81529.85 | 179.35 | -40759.93 |
| Snowmobile | Snmb_Trail1k*Area | 6 | 81564.13 | 213.63 | -40776.06 |
| Snowmobile | Snmb_Trail1k+Canopy | 4 | 81605.84 | 255.34 | -40798.92 |
| Snowmobile | Snmb_Intensity1k*Canopy | 5 | 81682.10 | 331.60 | -40836.00 |
| Snowmobile | Snmb_Intensity1k*Time + Canopy | 6 | 81686.31 | 335.81 | -40837.15 |
| Snowmobile | Snmb_Intensity1k + Canopy | 4 | 81689.60 | 339.10 | -40840.80 |
| Snowmobile | Canopy cover | 3 | 81766.20 | 415.70 | -40880.10 |
| Snowmobile | Snmb_Trail1k*Time | 5 | 95210.63 | 1827.00 | -41585.70 |
| Snowmobile | Snmb_Trail1k | 3 | 95225.86 | 13860.13 | -47600.31 |
| Snowmobile | Snmb_Intensity1k | 3 | 95391.61 | 13875.36 | -47609.93 |
| Snowmobile | Null | 2 | 95394.90 | 14041.11 | -47695.50 |
|  |  |  |  |  |  |
| Packed-trail Ski | PT-Ski_Trail500*Canopy | 5 | 80345.95 | 69.64 | -40168.00 |
| Packed-trail Ski | PT-Ski_Trail500*Area | 6 | 80465.46 | 189.15 | -40226.70 |
| Packed-trail Ski | PT-Ski_Trail500+Canopy | 4 | 80548.53  202.58 | 202.58 | -40270.26 |
| Packed-trail Ski | PT-Ski_Intensity1k*Canopy | 5 | 81364.70 | 1088.39 | -40677.30 |
| Packed-trail Ski | PT-Ski_Intensity1k*Area + Canopy | 6 | 81448.80 | 1172.49 | -40718.40 |
| Packed-trail Ski | PT-Ski_Intensity1k*Time + Canopy | 6 | 81453.22 | 1176.91 | -40720.60 |
| Packed-trail Ski | PT-Ski_Intensity1k + Canopy | 4 | 81459.10 | 1182.79 | -40725.60 |
| Packed-trail Ski | Canopy cover | 3 | 81766.20 | 1489.89 | -40880.10 |
| Packed-trail Ski | PT-Ski_Trail500 | 3 | 93326.41 | 12980.46 | -46660.21 |
| Packed-trail Ski | PT-Ski_Trail500*Time | 5 | 93330.11 | 12984.16 | -46660.05 |
| Packed-trail Ski | PT-Ski_Intensity1k | 3 | 94516.62 | 14170.67 | -47255.31 |
| Packed-trail Ski | Null | 2 | 95394.90 | 15118.59 | -47695.50 |

Appendix Table 3: Candidate models considered for proportion of time Canada lynx spent active versus stationary in response to temporal period for four types of winter recreation in western Colorado, USA, 2010-2013. Model parameters include time of day (TOD; day or night) and study area (Area; Vail or San Juan). Two candidate models for each recreation type were considered: an interaction of time of day with recreation intensity (TOD Interaction), and an interaction with study area plus time of day (Vail day, Vail night, San Juan day, San Juan night) with recreation intensity (Area+TOD Interaction). Within recreation type, models are ranked by AIC_c_, with the best performing listed first in bold. Type of recreation (Mode), number of model parameters (K), difference in AIC_c_ values (ΔAIC_c_), and model log likelihood (LL) are shown. The model including the effect of area was more supported in all cases.

| Mode | Model | K | AICc | ΔAICc | LL |
| --- | --- | --- | --- | --- | --- |
| Hybrid | **Area+TOD Interaction** | 9 | 47194.15 | 0.00 | -23588.07 |
| Hybrid | TOD Interaction | 5 | 47242.35 | 48.20 | -23616.17 |
| Hybrid | Null | 2 | 47282.60 | 88.45 | -23639.30 |
| Back-country Ski | **Area+TOD Interaction** | 9 | 47184.78 | 0 | -23583.4 |
| Back-country Ski | TOD Interaction | 5 | 47226.69 | 41.91 | -23608.3 |
| Back-country Ski | Null | 2 | 47282.60 | 97.82 | -23639.30 |
| Snowmobile | **Area+TOD Interaction** | 9 | 47176.31 | 0.00 | -23579.15 |
| Snowmobile | TOD Interaction | 5 | 47226.82 | 50.51 | -23608.41 |
| Snowmobile | Null | 2 | 47282.60 | 106.29 | -23639.30 |
| Packed-trail Ski | **Area+TOD Interaction** | 9 | 47127.53 | 0.00 | -23554.76 |
| Packed-trail Ski | TOD Interaction | 5 | 47179.02 | 51.49 | -23584.51 |
| Packed-trail Ski | Null | 2 | 47282.60 | 155.07 | -23639.30 |

Appendix Table 4: Candidate models predicting the presence of Canada lynx GPS locations inside ski area boundaries in western Colorado, USA, 2010-2013. Number of model parameters (K), difference in AIC_c_ values (ΔAIC_c_), and model log likelihood (LL) are shown. Model parameters include a continuous variable of Month, weekday/weekend (DOW), day/night (TOD), and percent canopy cover (Canopy). The top-performing model is given in bold.

| Model Structure | K | AIC_c_ | ΔAIC_c_ | LL |
| --- | --- | --- | --- | --- |
| **Month*DOW+TOD+Canopy** | 7 | 6724.74 | 0 | -3355.37 |
| Month+**DOW*****TOD**+ **Canopy** | 7 | 6738.43 | 13.69 | -3362.21 |
| Month***TOD**+**DOW**+ **Canopy** | 7 | 6744.68 | 19.94 | -3365.34 |
| Month+**TOD**+**DOW**+ **Canopy** | 6 | 6755.06 | 30.32 | -3371.53 |
| Month+**DOW**+ **Canopy** | 5 | 6757.33 | 32.59 | -3373.66 |
| Month+**TOD**+ **Canopy** | 5 | 6777.96 | 53.22 | -3383.98 |
| Month+ **Canopy** | 4 | 6780.19 | 55.45 | -3386.09 |
| **DOW*****TOD**+ **Canopy** | 6 | 6814.1 | 89.36 | -3401.05 |
| **DOW**+ **Canopy** | 4 | 6830.11 | 105.37 | -3411.05 |
| **Canopy** | 3 | 6853.32 | 128.57 | -3423.66 |
| **TOD**+ **Canopy** | 4 | 6853.86 | 129.11 | -3422.93 |
| Null | 2 | 6861.640 | 136.86 | -3428.82 |

Appendix Table 5: Summary statistics for recreation intensity on each Canada lynx’s yearly 95% minimum convex polygon home range (n=22) in western Colorado, USA, 2010-2013. For each lynx’s yearly home range (HR), the number of recreation tracks that we recorded of each type in home ranges is given (#), along with total length of track (km) of each type recorded in each home range, the home range size (km^2^), the density of all recreation tracks combined (linear km of recreation tracks/km^2^ home range area), and the average (Avg) and standard deviation (SD) of trail counter hits per day at all trail counters within each lynx’s home range.

| Study Area | Indv ID | # Recreation Tracks on HR | | | | km of Recreation Tracks on HR | | | | HR size (km^2^) | Track density km/km^2^ | Avg counter hits/day | SD counter hits/day |
| --- | --- | --- | --- | --- | --- | --- | --- | --- | --- | --- | --- | --- | --- |
|  |  | Hyb | BKSki | Snmb | PTSki | Hyb | BCski | Snmb | PTSki |  |  |  |  |
| Vail | Stafford Female 2011 | 134 | 27 | 126 | 2 | 1182 | 170 | 1079 | 17 | 31.37 | 78.00 | 53.11 | 47.29 |
| Vail | Turquoise Female 2013 | 4 | 13 | 25 | 24 | 32 | 44 | 300 | 100 | 45.03 | 10.57 | 65.67 | 0.0 |
| Vail | Stafford Female 2010 | 0 | 0 | 2 | 0 | 0 | 0 | 11 | 0 | 14.72 | 0.74 | 86.27 | 74.61 |
| Vail | Breckenridge Female 2011 | 0 | 3 | 0 | 1 | 0 | 12 | 0 | 4 | 34.56 | 0.47 | 16.83 | 5.94 |
| Vail | Breckenridge Female 2010 | 0 | 0 | 0 | 0 | 0 | 0 | 0 | 0 | 25.07 | 0.00 | 0.00 | 0.0 |
| Vail | Climax Female 2010 | 0 | 0 | 0 | 0 | 0 | 0 | 0 | 0 | 35.09 | 0.00 | 12.81 | 9.82 |
| Vail | Stafford Male 2011 | 132 | 24 | 123 | 3 | 494 | 167 | 435 | 21 | 92.14 | 12.11 | 24.16 | 28.42 |
| Vail | Turquoise Male 2013 | 4 | 20 | 25 | 29 | 41 | 59 | 410 | 157 | 135.43 | 4.93 | 46.85 | 25.07 |
| Vail | Half Moon Male 2013 | 4 | 14 | 25 | 18 | 23 | 41 | 163 | 66 | 175.41 | 1.67 | 34.38 | 25.33 |
| Vail | Climax Male 2011 | 0 | 17 | 0 | 2 | 0 | 41 | 0 | 6 | 85.06 | 0.55 | 18.69 | 12.33 |
| San Juan | Molas Female 2012 | 7 | 53 | 43 | 57 | 122 | 222 | 1660 | 215 | 106.77 | 20.79 | 11.21 | 4.86 |
| San Juan | Cement Female 2012 | 1 | 37 | 0 | 5 | 4 | 138 | 0 | 27 | 48.68 | 3.47 | 4.10 | 2.77 |
| San Juan | Hope Lake Female 2013 | 1 | 64 | 6 | 172 | 3 | 226 | 98 | 1118 | 67.36 | 21.45 | 30.25 | 22.07 |
| San Juan | Animas Female 2012 | 1 | 4 | 1 | 4 | 1 | 16 | 40 | 22 | 79.62 | 1.00 | 8.11 | 1.14 |
| San Juan | Ironton Female 2012 | 0 | 22 | 1 | 48 | 0 | 39 | 3 | 187 | 43.20 | 5.30 | 24.42 | 19.95 |
| San Juan | South Mineral Male 2012 | 15 | 107 | 95 | 116 | 415 | 483 | 3269 | 569 | 663.02 | 7.14 | 11.38 | 9.88 |
| San Juan | Cement Male 2012 | 2 | 102 | 3 | 8 | 20 | 328 | 10 | 40 | 104.52 | 3.80 | 5.87 | 7.57 |
| San Juan | Molas Male 2012 | 7 | 69 | 43 | 56 | 122 | 247 | 1660 | 226 | 157.63 | 14.31 | 10.81 | 4.85 |
| San Juan | Ophir Male 2012 | 2 | 184 | 29 | 70 | 35 | 883 | 508 | 267 | 175.51 | 9.64 | 29.10 | 19.80 |
| San Juan | Ironton Male 2012 | 0 | 22 | 1 | 48 | 0 | 35 | 3 | 186 | 42.26 | 5.32 | 27.45 | 16.08 |
| San Juan | South Mineral Male 2013 | 0 | 71 | 6 | 127 | 0 | 251 | 41 | 962 | 75.22 | 16.67 | 34.50 | 22.60 |
| San Juan | Ophir Male 2013 | 0 | 127 | 20 | 86 | 0 | 658 | 469 | 279 | 200.30 | 7.02 | 24.15 | 27.32 |

Appendix Table 6: Coefficients (β) and confidence intervals (95% CI) for proportion of time Canada lynx spent active versus stationary in response to an interaction of temporal period and study area (Vail day, Vail night, San Juan day, San Juan night) with recreation intensity at the 1km scale. Each recreation type was modeled separately, the reference group for each model was ‘San Juan day’. Covariates whose 95% CI did not overlap 0 are bolded. Model predictions are visualized in the manuscript in Figure 5.

|  | β | Lower 95% | Upper 95% |
| --- | --- | --- | --- |
| **Hybrid** |  |  |  |
| **San Juan night** | 0.12 | 0.06 | 0.18 |
| **Vail day** | -0.24 | -0.47 | -0.02 |
| Vail night | 0.19 | -0.04 | 0.41 |
| **Hybrid** | -0.68 | -1.06 | -0.3 |
| **San Juan night:Hybrid** | 0.75 | 0.31 | 1.2 |
| **Vail day:Hybrid** | 0.65 | 0.27 | 1.03 |
| **Vail night:Hybrid** | 0.67 | 0.29 | 1.05 |
| Random Effect | Var: 0.05 | SD: 0.23 |  |
| **Back-country Ski** |  |  |  |
| **San Juan night** | 0.07 | 0.02 | 0.12 |
| **Vail day** | -0.32 | -0.56 | -0.09 |
| Vail night | 0.14 | -0.09 | 0.37 |
| **Back-country Ski** | 0.08 | 0.05 | 0.12 |
| San Juan night:Ski | -0.03 | -0.08 | 0.01 |
| **Vail day:Ski** | **-0.18** | -0.32 | -0.04 |
| Vail night:Ski | -0.06 | -0.16 | 0.05 |
| Random Effect | Var: 0.06 | SD: 0.24 |  |
| **Snowmobile** |  |  |  |
| **San Juan night** | 0.06 | 0.02 | 0.11 |
| **Vail day** | -0.32 | -0.54 | -0.1 |
| Vail night | 0.12 | -0.09 | 0.34 |
| **Snowmobile** | 0.06 | 0.02 | 0.1 |
| San Juan night:Snmb | 0.03 | -0.02 | 0.08 |
| **Vail day:Snmb** | **-0.13** | -0.22 | -0.04 |
| Vail night:Snmb | -0.06 | -0.14 | 0.03 |
| Random Effect | Var: 0.05 | SD: 0.23 |  |
| **Packed-Trail Ski** |  |  |  |
| **San Juan night** | 0.06 | 0.01 | 0.1 |
| **Vail day** | -0.35 | -0.62 | -0.09 |
| **Vail night** | 0.26 | 0.01 | 0.51 |
| Packed-trail Ski | 0.03 | -0.01 | 0.06 |
| **San Juan night:PTSki** | **0.1** | 0.06 | 0.15 |
| Vail day:PTSki | -0.27 | -0.66 | 0.13 |
| **Vail night:PTSki** | **0.4** | 0.07 | 0.72 |
| Random Effect | Var: 0.06 | SD: 0.25 |  |
